# The effect of malaria on haemoglobin concentrations: a nationally representative household fixed-effects study of 17,599 children under 5 years of age in Burkina Faso

**DOI:** 10.1186/s12936-021-03948-z

**Published:** 2021-10-23

**Authors:** Tim Starck, Caroline A. Bulstra, Halidou Tinto, Toussaint Rouamba, Ali Sie, Thomas Jaenisch, Till Bärnighausen

**Affiliations:** 1 Heidelberg Institute of Global Health, Heidelberg University Medical Cente, Heidelberg, Germany; 2 Department of Public Health, Erasmus MC, University Medical Center Rotterdam, Rotterdam, The Netherlands; 3 Clinical Research Unit of Nanoro, Institut de Recherche en Sciences de la Santé, Nanoro, Burkina Faso; 4 National Institute of Public Health (INSP), Nouna Health Research Centre (CRSN), Nouna, Burkina Faso; 5 Center for Global Health, Colorado School of Public Health, Aurora, USA; 6 Department of Epidemiology, Colorado School of Public Health, Aurora, USA; 7 Harvard Center for Population and Development Studies, Boston, USA; 8 Africa Health Research Institute (AHRI), Durban, KwaZulu-Natal South Africa

**Keywords:** Malaria, Anaemia, Haemoglobin, Household fixed-effects, Burkina Faso, Rapid diagnostic tests, Microscopy

## Abstract

**Background:**

Although the association between malaria and anaemia is widely studied in patient cohorts, the population-representative causal effects of malaria on anaemia remain unknown. This study estimated the malaria-induced decrease in haemoglobin levels among young children in malaria-endemic Burkina Faso.

**Methods:**

The study was based on pooled individual-level nationally representative health survey data (2010–2011, 2014, 2017–2018) from 17 599 children under 5 years of age. This data was used to estimate the effects of malaria on haemoglobin concentration, controlling for household fixed-effects, age, and sex in a series of regression analyses. The fixed-effects controlled for observed and unobserved confounding on the household level and allowed to determine the impact of malaria infection status on haemoglobin levels and anaemia prevalence. Furthermore, the diagnostic results from microscopy and rapid diagnostic tests were leveraged to provide a quasi-longitudinal perspective of acute and prolonged effects after malaria infection.

**Results:**

The prevalence of both malaria (survey prevalence ranging from 17.4% to 65.2%) and anaemia (survey prevalence ranging from 74% to 88.2%) was very high in the included surveys. Malaria was estimated to significantly reduce haemoglobin levels, with an overall effect of − 7.5 g/dL (95% CI − 8.5, − 6.5). Acute malaria resulted in a − 7.7 g/dL (95% CI − 8.8, − 6.6) decrease in haemoglobin levels. Recent malaria without current parasitaemia decreased haemoglobin concentration by − 7.1 g/dL (95% CI − 8.3, − 5.9). The in-sample predicted prevalence of severe anaemia was 9.4% among malaria positives, but only 2.2% among children without malaria.

**Conclusion:**

Malaria infection has a strong detrimental effect on haemoglobin levels among young children in Burkina Faso. This effect seems to carry over even after acute infection, indicating prolonged haemoglobin reductions even after successful parasite-elimination. The quasi-experimental fixed-effect approach adds a population level perspective to existing clinical evidence.

**Supplementary Information:**

The online version contains supplementary material available at 10.1186/s12936-021-03948-z.

## Background

Burkina Faso has, according to World Health Organization (WHO) data, by far the highest prevalence of anaemia worldwide among young children (86% in 2016) [1]. It is also among the countries in sub-Saharan Africa that are burdened the most by malaria [2]. A large portion (43.8%) of Burkinabe people live in conditions marked by extreme poverty (less than 1.90 USD a day) and they and their children are thus particularly vulnerable to anaemia, malaria, and the associated, serious long term health consequences [3, 4]. However, Burkina Faso was also the setting of a recent, highly promising malaria-vaccine trial [5].

Malaria is a disease, caused by the *Plasmodium* spp. parasites that replicate in the human liver and erythrocytes [6]. In 2019, malaria accounted for the deaths of 384,000 children younger than 5 years in sub-Saharan Africa, 4% of which occurred in Burkina Faso [2]. This high death toll is partially a consequence of acute anaemia, one of malaria’s most prominent complications. However, malarial anaemia can also become chronic through pathways of persistent inflammation and bone marrow suppression leading to reduced production of erythrocytes [7–9]. Since anaemic children have lower oxygen-capacity, they are more susceptible to opportunistic infections, more tired, and less resilient than healthy children [10]. These symptoms ultimately add up to a higher risk of cognitive and physical development deficits in anaemic young children [3, 10–14].

Anaemia is screened for by measuring blood haemoglobin (Hb [g/L]), the erythrocytic protein that binds oxygen. Although the effect of malaria on haemoglobin has been the subject of extensive clinical research, epidemiological investigations on the population impact are severely lacking. The difficulty with determining the malaria attributable effect to population-wide haemoglobin reductions lies in the complex aetiology of anaemia. Aside from malaria, anaemia in children can be caused by genetic predisposition, other infectious diseases and wider socio-economic factors, especially nutritional deficits [10]. These contributors to anaemia are difficult to measure and may vary substantially between and even within given countries, thus making it hard to causally determine the impact of any one particular contributor to anaemia on the population level.

**Fig. 1:**
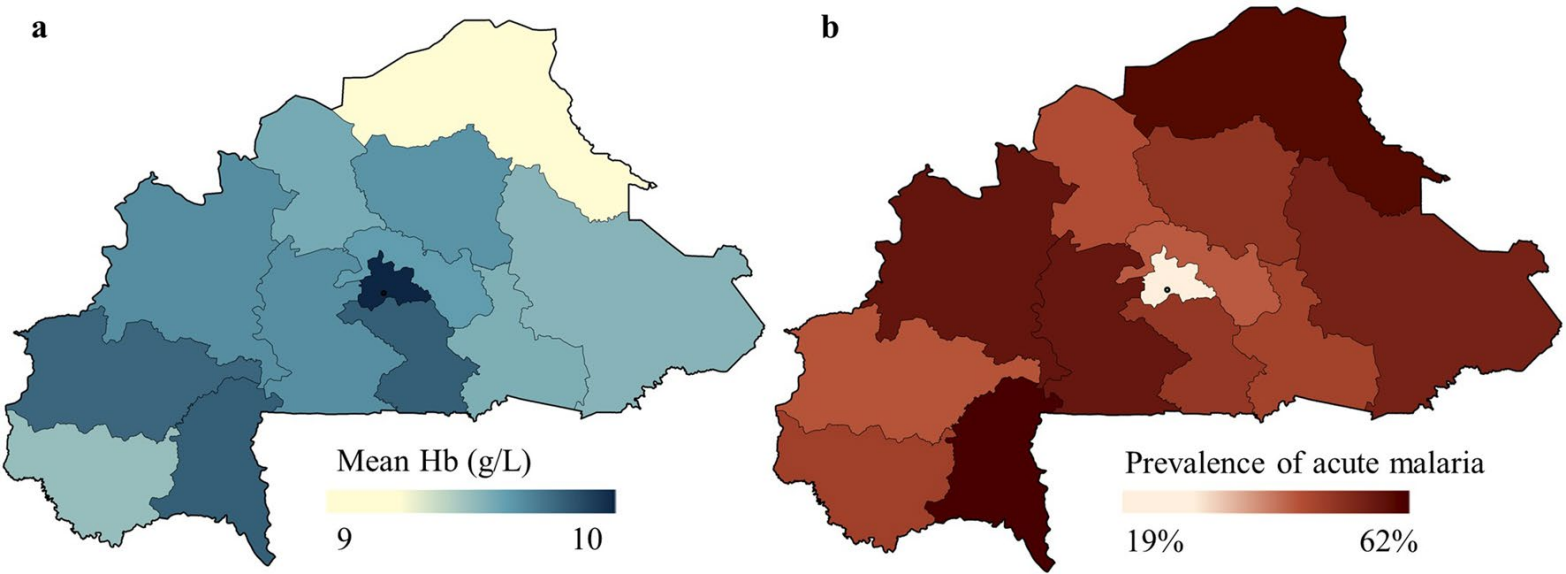
Map of the mean regional haemoglobin (**a**) and prevalence of malaria (**b**) in Burkina Faso. Panel **a** indicates the pooled mean regional haemoglobin values across all three surveys. Lighter colours represent lower average haemoglobin values and thus higher prevalence of anaemia. Panel **b** indicates the pooled mean regional prevalence of acute malaria as diagnosed by thick smear microscopy. Darker colours represent higher prevalence of anaemia. Demographic and Health Survey (DHS) and Malaria Indicator Survey (MIS) data obtained through https://dhsprogram.com/

Most epidemiologic studies that try to causally link malaria to population haemoglobin are conducted within relatively small local communities within highly endemic areas [9, 15–18]. They consistently report a detrimental effect over time of acute and repeated malaria infections on haemoglobin levels among children and adults [19–21]. While this important research confirms the causal effect of malaria on anaemia, external validity of these studies is often low due to regional restrictions and comparatively small or non-representative study populations. This study aims to build on this base by using an econometric modelling approach, fixed-effect analysis, that makes it possible to approximate causal effects by implicitly controlling for known and unknown confounders at the household level [22–25]. The household fixed-effect analysis is an extension on the analytic concept of repeated measures data, where every individual within a household is considered a measurement of the same entity, *i.e.,* the household. This study applies the fixed-effect approach to a large cross-sectional and nationally representative dataset of young children in Burkina Faso, a country burdened by extreme prevalence of malaria and anaemia alike. Burkina Faso is therefore a highly relevant target in the effort to combat both (Fig. 1).

This study aims to contribute to the fight against the harmful effects of anaemia in Burkina Faso by providing a better understanding of the contribution of malaria to the burden of anaemia. The quasi-experimental study design makes it possible to estimate the population-wide effect of malaria infection on haemoglobin levels in children, while avoiding ethically questionable randomized controlled study designs. This will also underline the importance of eliminating malaria and illustrates the potential gains from the recent vaccine candidate in Burkina Faso [5].

## Methods

### Data

The analysis is based on pooled data from the 2010 Demographic and Health Survey (DHS) and 2014 and 2017–18 Malaria Indicator Surveys (MISs) from Burkina Faso. The DHSs and MISs are nationally representative household surveys that include demographic, health, and nutrition data, including recognized malaria indicators. The combined datasets contained data from 17,599 children from 11,886 households divided over 572 sample clusters. Field operators collected the survey data from May 2010 to January 2011 (DHS 2010–2011), from September to October 2014 (MIS 2014) and from November 2017 to March 2018 (MIS 2017–2018). Malaria transmission in Burkina Faso is highest from July to November, therefore the pooled surveys reflect the annual average alongside changes between years, e.g., successful implementation of malaria programmes [26–28]. New households were selected for each survey; therefore, this study does not offer a longitudinal perspective on the individual households. During the surveys, field workers gathered data for two parallel methods of malaria diagnostics: rapid diagnostic antigen tests (RDT) for immediate screening results and thick smear microscopy (henceforth referred to as microscopy) to be analysed later in central laboratories. The RDT model for MIS 2014 and 2017–2018 was “SD Bioline Pan/Pf” (manufacturer: Standard Diagnostics, Inc.), a combined HRP-2/pLDH-test. The MIS 2010 final report did not specify the RDT model or type. Haemoglobin concentrations (g/L, Hb) were collected with the survey and measured by HemoCue test (manufacturer: HemoCue). More information on the data and collection process can be found at www.dhsprogram.com.

The following variables were extracted from the surveys for analysis: RDT results, microscopy test result, haemoglobin blood levels, age in months, sex, and household identifier. The severity of anaemia was categorized according to the WHO cut-off values for anaemia among children 59 and younger: any haemoglobin concentrations of less than 110 g/L were considered anaemia, further classified as mild (90–109 g/L), moderate (70–89 g/L) or severe anaemia (< 70 g/L) [29]. Age in months was converted to age groups by completed years (6–12; 13–24; 25–36; 37–48; 49–59 months).

The presence of *Plasmodium* spp. in the bloodstream was detected via thick smear microscopy, the current gold standard. Formally, ruling out malaria with microscopy requires three consecutive negative samples. Therefore, since survey samples were only collected once, DHS microscopy results are at risk of false negatives. Rapid diagnostic tests detect *Plasmodium* antigens in the blood stream for the diagnosis of malaria infections. These antigens have been reported to remain positive for up to 30 days after an acute is treated or controlled [30–32]. This results in a time window, where positive microscopy represents acute cases and positive RDTs with negative microscopy indicate post-infection status [33]. This time-lage was exploited to create a quasi-longitudinal perspective in the cross-sectional data to illustrate acute and prolonged effects of malaria on haemoglobin. Malaria status was further stratified into three separate groups for analysis, firstly “overall malaria” including all cases with any positive malaria test (RDT or microscopy), secondly “acute malaria” which included all cases that tested positive in microscopy screening and thirdly “sub-microscopic malaria” which included only those cases, that tested positive by RDT but negative by microscopy. A sub-microscopic infection refers to a time window either before or after the acute infection crosses the microscopy detection threshold and in addition to the time window when remaining circulating antigens cause the RDT to be positive after the acute infection has been cleared. Within this period, the patient is usually asymptomatic, but can be symptomatic in rare cases.

### Statistical methods

The primary analysis estimated the population-level effect of malaria on haemoglobin concentrations (g/L) in a series of nested linear regression models. The first model, the overall malaria model, used any positive malaria-test result (RDT or microscopy) as indicator of malaria infection. The second, the stratified model, used the two malaria measurements, microscopy and RDT, to create three malaria-status groups: malaria negative (if microscopy and RDT negative), acute malaria (if microscopy positive), sub-microscopic malaria (if microscopy negative, but RDT positive). The (econometric) household fixed-effects were included in all primary analyses to control for observed and unobserved confounders that are shared between all children within one given household [22, 23]. These confounders include known factors such as socioeconomic, temporal, and spatial differences that vary between households but not within. Unknown factors could, amongst others, include nutritional or socio-economic traits which are likely shared between family members within one household but not across households [10].

The final model was stratified for malaria status, age, sex and household fixed-effect. For the mathematical formula of the final model please refer to Additional file 1: S29. Survey weights were not applied because the within-survey weights are not representative for multiple-survey analyses.

To provide a more tangible perspective on the malaria-attributable effect on anaemia prevalence in the observed population, in-sample predictions were generated to illustrate the possible changes in anaemia prevalence by eliminating malaria. The predictions were calculated based on the indidividual haemoglobin estimates from each child in the overall malaria and stratified models. For the predictions, anaemia was stratified into three clinically relevant severity groups (any anaemia if Hb < 110 g/L; moderate or worse anaemia if Hb < 90 g/L; severe anaemia if Hb < 70 g/L) to assess the influence of malaria on anaemia prevalence by severity.

Finally, an additional series of sensitivity analyses on the stratified model were performed to further validate the modelling approach. Firstly, the nested series was repeated to check for possible interaction between malaria, age, and sex, respectively and combined. Secondly, subgroup analyses were added based on sex, survey, and malaria season. An additional nested series with household modelled as random rather than fixed effect concluded the sensitivity analyses. The random-effects modelwas expected to show larger effects than the corresponding fixed-effects model.

All analyses were done in R version 4.0.2 or higher (“plm” package version 2.2-5, “fixest” package version 0.8.2). Maps were generated with ArcGIS Pro version 2.3.

## Results

**Fig. 2:**
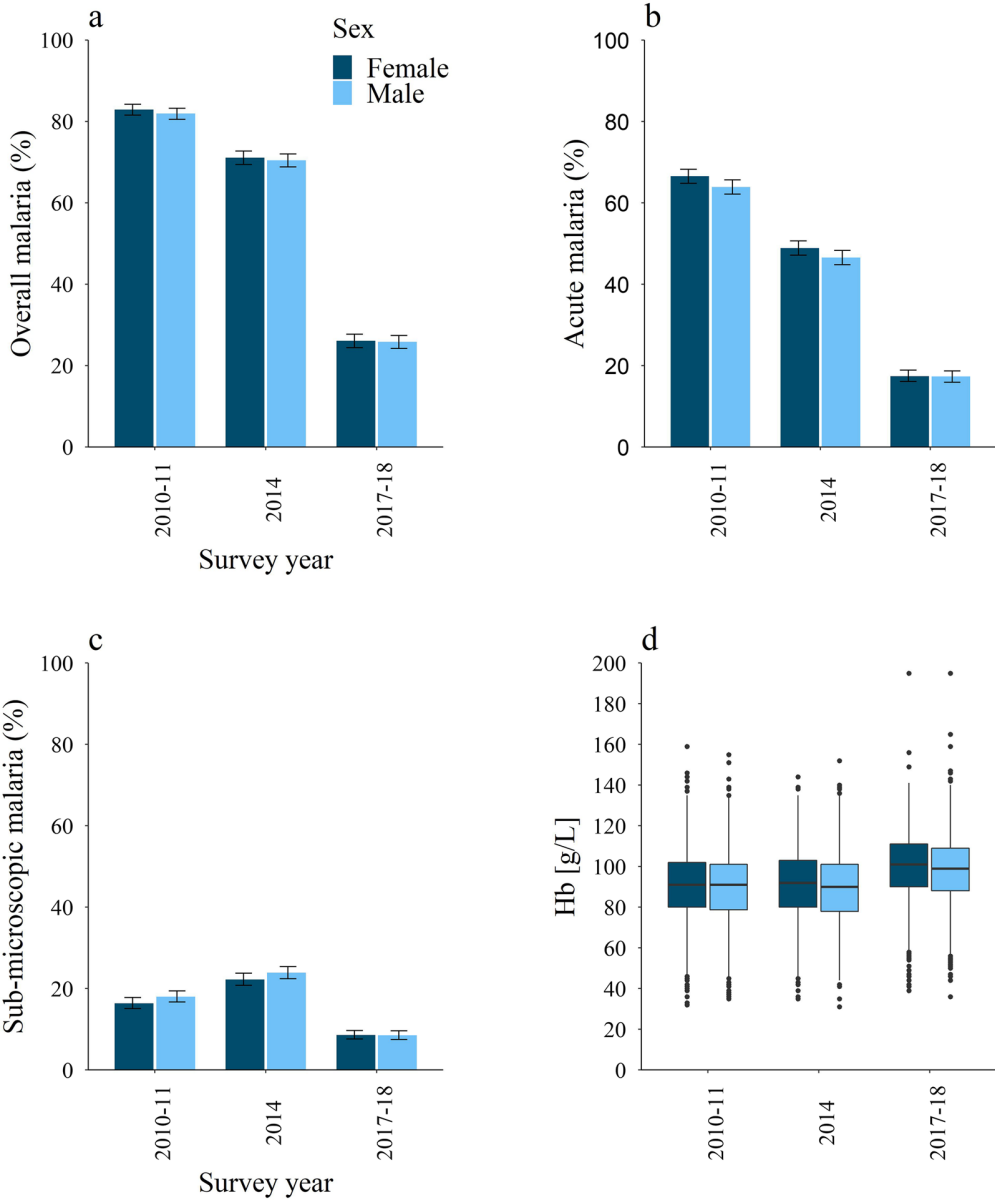
Prevalence of overall (**a**), acute (**b**) and sub-microscopic malaria (**c**); Haemoglobin concentrations [g/L] (**d**). The plots demonstrate the change in the study populations from the successive surveys based on sex. The error bars mark the 95% confidence intervals in panels **a**, **b** and **c**. Dark blue (dark grey, left column) represents females, light blue (grey, right column) represents males. Demographic and Health Survey (DHS) and Malaria Indicator Survey (MIS) data obtained through https://dhsprogram.com/

A summary of the population and survey characteristics is provided in Table 1. The final sample included 17 599 children from 11 816 households in Burkina Faso, aged 6 months to 5 years. Figure 2 and Table 1 present a more detailed description of malaria and anaemia in the study populations by survey. The prevalence of malaria varied between the consecutive surveys and averaged 44% for the pooled data (17.4–65.2%, Table 1). Anaemia (haemoglobin < 110 g/L) prevalence showed less variation between surveys but also declined over the years. The overall anaemia prevalence for the pooled data was 83.2%, 31.1% for moderate anaemia and 9.2% for severe anaemia. Regional distributions of malaria prevalence and average haemoglobin levels across the pooled surveys are shown in Fig. 1. An additional plot of the haemoglobin levels by malaria status, age, and sex was added to the Additional file 1: S30. The differences in mean haemoglobin for the subgroups of negative malaria and acute malaria [p < 0.001], negative malaria and sub-microscopic malaria [p < 0.001] and acute and sub-microscopic malaria [p < 0.001] were statistically significant in unpaired T-tests.

**Table 1:** Characteristics of study population and subgroups

	Overall (pooled data sample)	Stratified by survey			Stratified by sex	
		2010–2011 May–Jan^a^	2014 Sept–Dec^a^	2017–2018 Dec–March	Males	Females
Sample size	17,599	5926	6110	5563	8962	8637
Sex (%)						
Female	8637 (49.1)	2882 (48.6)	3003 (49.1)	2752 (49.5)	–	–
Male	8962 (50.9)	3044 (51.4)	3107 (50.9)	2811 (50.5)	–	–
Age [months] (%)						
6–12	1918 (10.9)	651 (11)	657 (10.8)	610 (11)	984 (11)	934 (10.8)
13–24	3750 (21.3)	1301 (22)	1297 (21.2)	1152 (20.7)	1945 (21.7)	1805 (20.9)
25–36	3942 (22.4)	1328 (22.4)	1404 (23)	1210 (21.8)	2032 (22.7)	1910 (22.1)
37–48	4091 (23.2)	1363 (23)	1402 (22.9)	1326 (23.8)	2020 (22.5)	2071 (24)
49–59	3898 (22.1)	1283 (21.7)	1350 (22.1)	1265 (22.7)	1981 (22.1)	1917 (22.2)
Malaria status (%)						
Negative	6954 (39.5)	1045 (17.6)	1787 (29.2)	4122 (74.1)	3558 (39.7)	3396 (39.3)
Acute	7740 (44)	3861 (65.2)	2913 (47.7)	966 (17.4)	3876 (43.2)	3864 (44.7)
Sub-microscopic	2905 (16.5)	1020 (17.2)	1410 (23.1)	475 (8.5)	1528 (17)	1377 (15.9)
Anaemia						
Mean Hb (g/L) (SD)	93 (17)	90 (17)	90 (17)	99 (17)	92 (17)	94 (17)
Anaemic	14 639 (83.2)	5229 (88.2)	5291 (86.6)	4119 (74)	7576 (84.5)	7063 (81.8)
Not anaemic	2960 (16.8)	697 (11.8)	819 (13.4)	1444 (26)	1386 (15.2)	1574 (18.2)
Mild	7535 (42.8)	2464 (41.6)	2382 (39)	2689 (48.3)	3802 (42.4)	3733 (43.2)
Moderate	5482 (31.1)	2135 (36)	2112 (34.6)	1235 (22.2)	2856 (31.9)	2626 (30,4)
Severe	1622 (9.2)	630 (10.6)	797 (13.0)	195 (3.5)	918 (10.2)	704 (8.2)

Anaemia categories: Not anaemic (Hb ≥ 110 g/L); Mild anaemia (Hb < 110 g/L); Moderate anaemia (Hb < 90 g/L); Severe anaemia (Hb < 70 g/L) [29]. Due to rounding the percent might not add up to 100

*Hb* haemoglobin, *SD* standard deviation, *TSM *thick smear microscopy, *RDT* rapid diagnostic test

^a^Time frames covering malaria peak season in Burkina Faso from July to November

The outcomes of the overall malaria and stratified models and the respective reductions in haemoglobin are summarised in Table 2. In the overall model, a positive malaria test (RDT or microscopy) reduced haemoglobin by − 7.5 g/L [95% CI − 8.5; − 6.5]. In the stratified model (by malaria infection duration), acute malaria resulted in a − 7.7 g/L [95% CI − 8.8; − 6.6] decrease in haemoglobin concentration after controlling for age and sex. The prolonged effect after a cleared malaria infection was − 7.1 g/L [95% CI − 8.3; − 5.9] (Additional file 1: S30). Older age had an increasingly beneficial effect on haemoglobin levels, except for the 12–24 months age-group, that conversely had reduced haemoglobin levels of − 2 g/L [95% CI − 3.3; − 0.7]. Female sex had a protective effect of 2 g/L [95% CI 1.3; 2.7]. The results for the remaining models of the nested series were appended to the Additional file 1: S1–S8.

**Table 2:** Main household fixed-effect regression models of malaria status adjusted for sex and age

Coefficients		Overall malaria model^a^	Stratified model^b^
Malaria effect on haemoglobin			
Overall malaria status			
Reference: negative			
Malaria	Estimate	− 7.5	
	p-value	< 0.001	
	CI 95	(− 8.5; − 6.5)	
Stratified malaria status			
Reference: negative			
Acute			− 7.7
			< 0.001
			(− 8.8; − 6.6)
Sub-microscopic			− 7.1
			< 0.001
			(− 8.3; − 5.9)
Age group			
Reference:6–12 months			
13–24 months		− 2	− 2
		0.002	0.002
		(− 3.3; − 0.7)	(− 3.3; − 0.7)
25–36 months		2.8	2.8
		< 0.001	< 0.001
		(1.5; 4.1)	(1.5; 4.1)
37–48 months		8.3	8.3
		< 0.001	< 0.001
		(7.1; 9.4)	(7.1; 9.4)
49–50 months		13.2	13.3
		< 0.001	< 0.001
		(12.1; 14.4)	(12.1; 14.5)
Sex			
Reference: male			
Female		0.2	0.2
		< 0.001	< 0.001
		(0.13; 0.27)	(0.13; 0.27)

*CI *confidence interval

^a^Linear regression of all malaria positive cases on haemoglobin

^b^Linear regression of acute and sub-microscopic malaria cases on haemoglobin

The overall effect of all positive malaria tests is − 7.5 g/L (overall malaria model). Children with acute malaria have − 7.7 g/L less haemoglobin when compared to the healthy population. The prolonged effect after infection is − 7.1 g/L when compared to the healthy population (stratified model)

**Fig. 3:**
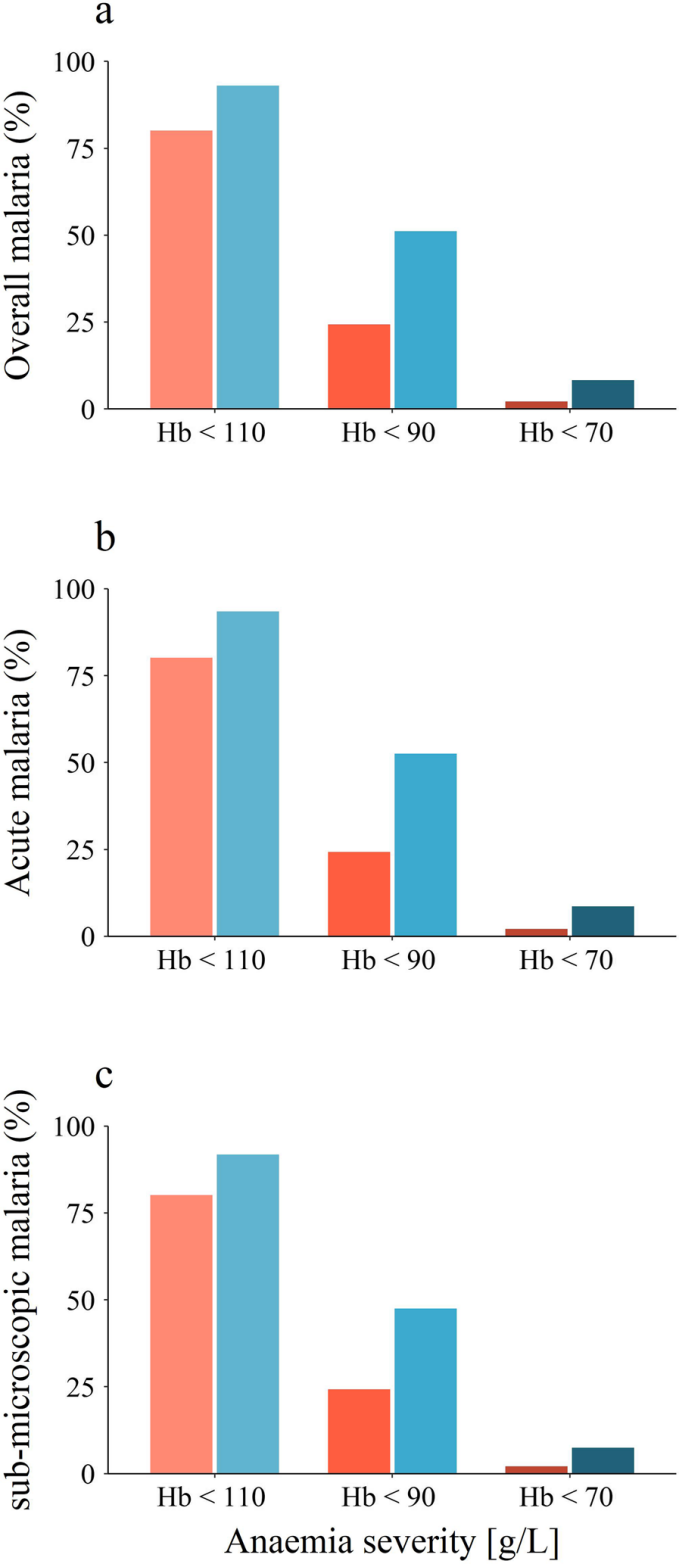
Predicted prevalence of any, moderate or worse and severe anaemia by malaria status. In-sample predicted prevalence of any anaemia (Hb < 110 g/L), moderate or worse anaemia (Hb < 90 g/L) and severe anaemia (Hb < 70 g/L) by malaria status for all malaria positive cases (**a**), acute cases (**b**) and sub-microscopic cases (**c**). Eliminating malaria from the study population would substantially reduce the total prevalence of malaria, moderate anaemia and almost eliminate severe anaemia. The stratification by malaria status affected the results only marginally. Red shades (lighter colours, left bars) indicate malaria negative cases and blue shades (darker colours, right bars) malaria positive cases

The in-sample predictions from the overall malaria model indicate a 92.5% prevalence of anaemia among malaria positive children, compared to 77.9% among malaria negative children. The absolute difference was largest for moderate or severe anaemia (Hb < 90 g/L; malaria positives: 51.5%, malaria negatives: 24.6%) and the relative difference was largest for severe anaemia (Hb < 70 g/L; malaria positives: 9.4%, malaria negatives: 2.2%). The results from the predictions are illustrated in Fig. 3.

The random effects models showed generally larger effects for the acute and prolonged effects (Additional file 1: S9–S15) but remained consistent with the results of the main fixed-effect models. The larger effects are attributable to the reduced control for confounding on the household level due to the random effect assumption and are thus likely the result of bias, which is eliminated in the household fixed-effect analysis. Similarly, the subset analysis for male participants (Additional file 1: S16–S18) and female participants (Additional file 1: S19–S21), seasonality (Additional file 1: S22, S23) and survey (Additional file 1: S24–S26) remained consistent with the main outcomes.

## Discussion

Both malaria prevalence (44% in the pooled data) and anaemia prevalence (83% in the pooled data) were high among young children in Burkina Faso between 2010 and 2018. This study estimated a malaria-attributable haemoglobin decrease of − 7.7 g/L during acute infection and of − 7.1 g/L in the time post-infection. Older children had higher haemoglobin levels than younger children and female sex improved haemoglobin levels by 2 g/L.

The malaria-induced haemoglobin changes can have large clinical implications. For instance, it has been shown that an increase of 10 g/L haemoglobin is associated with a 0.78 relative risk of mental retardation in young children [34]. This implies that a malaria-attributable haemoglobin reduction as shown in the data might pose substantial threat of cognitive development disorders in affected children. Especially the predictions of anaemia prevalence illustrate the severity of the burden of malarial anaemia in Burkina Faso and the analyses indicate that most cases of severe anaemia and a sizeable portion of moderate anaemia could be avoided if malaria were successfully eliminated.

Several studies have previously reported on the malaria-associated decrease in haemoglobin concentrations in clinical and national settings using different analytic methods [35–37]. The age group and sex dependent variation in haemoglobin values, as observed in this study, have been described previously in a similar fashion but especially the differences by sex might warrant further research [38].

This study is unique in that it could quantify the strong association between malaria and anaemia at the population-level, using a household fixed-effect approach controlling for all confounding that is constant within a given household. Furthermore, it is representative not only in its sampling design, but also in its seasonal composition, given that surveys were conducted on- and off malaria season. Finally, this study is based on a very large and nationally representative sample of 17,599 children and thus offers enough power to inspire confidence in its results as they are consistent even in the reduced subset analyses.

This study is influenced by several limitations. Firstly, a large number of children had a positive rapid test, but no corresponding positive microscopy test result. Thick smear microscopy is considered the gold standard but has varying sensitivity (from 55 to 98%) and specificity (from 81% to > 98%), depending on the experience of the diagnostician and the slide quality [39–41]. To rule out malaria it is required to repeat the microscopy test over the course of several days, which has not been done in the surveys and thus likely results in an underestimation of the malaria prevalence in this study [32]. RDTs on the other hand produce a comparatively high rate of false positives where *Plasmodium* antigens are present on the microscopically undetectable gametocytes, even when the disease itself is controlled by the immune system or medical treatment and parasitaemia is below the detection threshold. This can cause microscopy-negative cases to show RDT positive results for up to thirty days even after parasite elimination and clinical remission [30, 33]. The statistical model leveraged this effect to create a quasi-longitudinal perspective, where positive RDTs with negative microscopy results represent children who are currently recovering from malaria. Biologically, this prolonged effect might be a mix of several contributing factors, such as persistent bone-marrow suppression, delayed haemolysis, delayed recovery and false-negative microscopy tests [42].

A second limitation is the way in which the pooled cross-sectional data reflects the patterns of malaria and changes between survey years in Burkina Faso. Since several years and seasons of surveys were pooled for the analysis, the study population is not representative of any malaria point-prevalence in Burkina Faso and thus the data neither reflects malarias seasonal pattern, nor does it reflect progress made in the fight against malaria between 2010 and 2018. It is, however, still comparable to the extremes of anaemia burden and malaria transmission intensity found in West African countries [2, 43, 44].

Thirdly, the fixed-effect method itself also comes with a caveat. It controls for all confounders above the household level but lacks control for the within household confounders, particularly anaemia risk factors that vary between children in a household. These risk factors include nutritional (e.g., iron deficiency) and genetical traits (e.g., sickle-cell anaemia), other infectious diseases (e.g., helminths) and other, frequently interacted factors [45–47]. The modelling approach assumes that these unmeasured confounders are reasonably similar for all children within the household.

## Conclusions

In summary, this study proposes a strong estimate of the population-wide effect of malaria on haemoglobin among young children in Burkina Faso, a setting marked by high malaria burden and extremely high anaemia prevalence. The findings of this study shed light on the acute and prolonged effects of infection and the potential gains against severe anaemia by eliminating malaria. Hopefully, these effects will soon be diminished by effective, accessible and affordable malaria control strategies. The household fixed-effects analysis has proven to be a suitable design to quantify these effects in a quasi-experimental setup and makes it possible to draw strong conclusions. Further research should attempt to clarify how genetic relationship and other factors contribute to the risk of acquiring malaria infection and decreased haemoglobin levels. There is a large knowledge gap on the longitudinal change in haemoglobin levels during an acute malaria infection.

## Supplementary Information

**Additional file 1.** Online only supplement. Additional information, explanations and results published only only (S1–S30).

## Data Availability

All data is available from the DHS website (https://dhsprogram.com/). Codes will be made available upon request.

## Abbreviations

DHS: Demographic and health surveys
Hb: Haemoglobin
MIS: Malaria indicator surveys
RDT: Rapid diagnostic test
SSA: Sub-Saharan Africa
