# Supplementary material for: The effect of malaria on haemoglobin concentrations: a nationally representative household fixed-effects study of 17,599 children under 5 years of age in Burkina Faso
Source: Malar J. 2021 Oct 23;20:416. doi: 10.1186/s12936-021-03948-z (PMC8542337; doi:10.1186/s12936-021-03948-z)
Supplement: Supplementary file 1 — Additional file 1. Online only supplement. Additional information, explanations and results published only only (S1–S30). [file 12936_2021_3948_MOESM1_ESM.docx]

# Additional file 1

**S1 Table. Household fixed-effect regression model of malaria status.** Number of households is 11 886, prevalence of malaria is 44% and prevalence of anemia is 83.2%.

|  | **Effect of malaria on hemoglobin** | | | |
| --- | --- | --- | --- | --- |
| **Covariate** | N | Mean Hb  [SD] [g/L] | Hb Change  [95% CI] [g/L] | P-Value |
| **Baseline total** | 17 599 | 93 [17] | Reference |  |
| **Acute effect** | 7740 | 89 [17] | -4 [-6; -3] | < 0.001 *** |
| **Prolonged effect** | 2905 | 90 [17] | -5 [-7; -4] | 0.002 ** |

Significance codes: < 0.001 ‘***’; < 0.01 ‘**’; < 0.05 ‘*’

**S2 Table. Household fixed-effect regression model of malaria status adjusted for age.** Number of households is 11 886, prevalence of malaria is 44% and prevalence of anemia is 83.2%.

|  | **Effect of malaria on hemoglobin** | | | |
| --- | --- | --- | --- | --- |
| **Covariate** | N | Mean Hb  [SD] [g/L] | Hb Change  [95% CI] [g/L] | P-Value |
| **Baseline total** | 17 599 | 93 [17] | Reference |  |
| **Acute effect** | 7740 | 89 [17] | -8 [-9; -7] | < 0.001 *** |
| **Prolonged effect** | 2905 | 90 [17] | -7 [-8; -6] | < 0.001 *** |
| **Age** |  |  |  |  |
| 6 - 12 | 1918 | 88 [16] | Reference |  |
| 13 - 24 | 3750 | 87 [17] | -2 [-3; -1] | 0.003 ** |
| 25 – 36 | 3942 | 91 [17] | 3 [2; 4] | < 0.001 *** |
| 37 – 48 | 4091 | 96 [16] | 8 [7; 9] | < 0.001 *** |
| 49 – 59 | 3898 | 110 [15] | 13 [12; 14] | < 0.001 *** |

Significance codes: < 0.001 ‘***’; < 0.01 ‘**’; < 0.05 ‘*’

**S3 Table. Household fixed-effect regression model of malaria status adjusted for sex.** Number of households is 11 886, prevalence of malaria is 44% and prevalence of anemia is 83.2%.

|  | **Effect of malaria on hemoglobin** | | | |
| --- | --- | --- | --- | --- |
| **Covariate** | N | Mean Hb  [SD] [g/L] | Hb Change  [95% CI] [g/L] | P-Value |
| **Baseline total** | 17 599 | 93 [17] | Reference |  |
| **Acute effect** | 7740 | 89 [17] | -4 [-6; -3] | < 0.001 *** |
| **Prolonged effect** | 2905 | 90 [17] | -5 [-7; -4] | < 0.001 *** |
| **Sex** |  |  |  |  |
| Male | 8962 | 92 [17] | Reference |  |
| Female | 8637 | 94 [17] | 2 [1; 3] | < 0.001 *** |

Significance codes: < 0.001 ‘***’; < 0.01 ‘**’; < 0.05 ‘*’

**S4 Table. Main household fixed-effect regression model of malaria status adjusted for sex and age.** Number of households is 11 886, prevalence of malaria is 44% and prevalence of anemia is 83.2%.

|  | **Effect of malaria on hemoglobin** | | | |
| --- | --- | --- | --- | --- |
| **Covariate** | N | Mean Hb  [SD] [g/L] | Hb Change  [95% CI] [g/L] | P-Value |
| **Baseline total** | 17 599 | 93 [17] | Reference |  |
| **Acute effect** | 7740 | 89 [17] | -8 [-9; -7] | < 0.001 *** |
| **Prolonged effect** | 2905 | 90 [17] | -7 [-8; -6] | < 0.001 *** |
| **Sex** |  |  |  |  |
| Male | 8962 | 92 [17] | Reference |  |
| Female | 8637 | 94 [17] | 2 [1; 3] | < 0.001 *** |
| **Age** |  |  |  |  |
| 6 - 12 | 1918 | 88 [16] | Reference |  |
| 13 - 24 | 3750 | 87 [17] | -2 [-3; -1] | 0.002 ** |
| 25 – 36 | 3942 | 91 [17] | 3 [2; 4] | < 0.001 *** |
| 37 – 48 | 4091 | 96 [16] | 8 [7; 9] | < 0.001 *** |
| 49 – 59 | 3898 | 110 [15] | 13 [12; 14] | < 0.001 *** |

Significance codes: < 0.001 ‘***’; < 0.01 ‘**’; < 0.05 ‘*’

**S5 Table. Household fixed-effect regression model of malaria status adjusted for age and controlling for interaction between age and malaria status.** Number of households is 11 886, prevalence of malaria is 44% and prevalence of anemia is 83.2%.

|  | **Effect of malaria on hemoglobin** | | | |
| --- | --- | --- | --- | --- |
| **Covariate** | N | Mean Hb  [SD] [g/L] | Hb Change  [95% CI] [g/L] | P-Value |
| **Baseline total** | 17 599 | 93 [17] | Reference |  |
| **Acute effect** | 7740 | 89 [17] | -6 [-9; -4] | < 0.001 *** |
| **Prolonged effect** | 2905 | 90 [17] | -6 [-9; -3] | < 0.001 *** |
| **Age** |  |  |  |  |
| 6 - 12 | 1918 | 88 [16] | Reference |  |
| 13 - 24 | 3750 | 87 [17] | 0 [-2; 2] | 0.725 |
| 25 – 36 | 3942 | 91 [17] | 5 [3; 7] | < 0.001 *** |
| 37 – 48 | 4091 | 96 [16] | 9 [7; 11] | < 0.001 *** |
| 49 – 59 | 3898 | 110 [15] | 12 [10; 14] | < 0.001 *** |
| **Age * Malaria acute** |  |  |  |  |
| 6 – 12 |  |  | Reference |  |
| 13 – 24 |  |  | -5 [-7; -2] | 0.001 ** |
| 25 – 36 |  |  | -3 [-6; 0] | 0.031 * |
| 37 – 48 |  |  | 0 [-3; 2] | 0.734 |
| 49 – 59 |  |  | 2 [-1; 4] | 0.177 |
| **Age * Malaria sub-microscopic** |  |  |  |  |
| 6 – 12 |  |  | Reference |  |
| 13 – 24 |  |  | -2 [-6; 1] | 0.218 |
| 25 – 36 |  |  | -2 [-6; 1] | 0.205 |
| 37 – 48 |  |  | -2 [-5; 2] | 0.279 |
| 49 – 59 |  |  | 1 [-3; 4] | 0.832 |

Significance codes: < 0.001 ‘***’; < 0.01 ‘**’; < 0.05 ‘*’

**S6 Table. Household fixed-effect regression model of malaria status adjusted for sex and controlling for interaction between sex and malaria status.** Number of households is 11 886, prevalence of malaria is 44% and prevalence of anemia is 83.2%.

|  | **Effect of malaria on hemoglobin** | | | |
| --- | --- | --- | --- | --- |
| **Covariate** | N | Mean Hb  [SD] [g/L] | Hb Change  [95% CI] [g/L] | P-Value |
| **Baseline total** | 17 599 | 93 [17] | Reference |  |
| **Acute effect** | 7740 | 89 [17] | -4 [-5; -2] | < 0.001 *** |
| **Prolonged effect** | 2905 | 90 [17] | -4 [-6; -3] | < 0.001 *** |
| **Sex** |  |  |  |  |
| Male | 8962 | 92 [17] | Reference |  |
| Female | 8637 | 94 [17] | 3 [2; 4] | < 0.001 *** |
| **Sex * Malaria acute** |  |  |  |  |
| Male |  |  | Reference |  |
| Female |  |  | -1 [-3; 1] | 0.185 |
| **Sex * Malaria sub-microscopic** |  |  |  |  |
| Male |  |  | Reference |  |
| Female |  |  | -2 [-4; 0] | 0.095 |

Significance codes: < 0.001 ‘***’; < 0.01 ‘**’; < 0.05 ‘*’

**S7 Table. Household fixed-effect regression model of malaria status adjusted for age, sex, controlling for interaction between age and malaria status and sex and malaria status.** Number of households is 11 886, prevalence of malaria is 44% and prevalence of anemia is 83.2%.

|  | **Effect of malaria on hemoglobin** | | | |
| --- | --- | --- | --- | --- |
| **Covariate** | N | Mean Hb  [SD] [g/L] | Hb Change  [95% CI] [g/L] | P-Value |
| **Baseline total** | 17 599 | 93 [17] | Reference |  |
| **Acute effect** | 7740 | 89 [17] | -6 [-8; -4] | < 0.001 *** |
| **Prolonged effect** | 2905 | 90 [17] | -5 [-8; -2] | 0.002 ** |
| **Sex** |  |  |  |  |
| Male | 8962 | 92 [17] | Reference |  |
| Female | 8637 | 94 [17] | 3 [2; 4] | < 0.001 *** |
| **Age** |  |  |  |  |
| 6 - 12 | 1918 | 88 [16] | Reference |  |
| 13 - 24 | 3750 | 87 [17] | 0 [-2; 2] | 0.908 |
| 25 – 36 | 3942 | 91 [17] | 4 [2; 6] | < 0.001 *** |
| 37 – 48 | 4091 | 96 [16] | 8 [7; 10] | < 0.001 *** |
| 49 – 59 | 3898 | 110 [15] | 12 [10; 14] | < 0.001 *** |
| **Age * Malaria acute** |  |  |  |  |
| 6 – 12 |  |  | Reference |  |
| 13 – 24 |  |  | -4 [-7; 2] | 0.002 ** |
| 25 – 36 |  |  | -2 [-6; 0] | 0.056 |
| 37 – 48 |  |  | 0 [-3; 3] | 0.946 |
| 49 – 59 |  |  | 2 [-1; 5] | 0.136 |
| **Age * Malaria sub-microscopic** |  |  |  |  |
| 6 – 12 |  |  | Reference |  |
| 13 – 24 |  |  | -2 [-5; 2] | 0.284 |
| 25 – 36 |  |  | -2 [-6; 1] | 0.249 |
| 37 – 48 |  |  | -2 [-5; 2] | 0.343 |
| 49 – 59 |  |  | 1 [-3; 4] | 0.716 |
| **Sex * Malaria acute** |  |  |  |  |
| Male |  |  | Reference |  |
| Female |  |  | -1 [-3; 0] | 0.141 |
| **Sex * Malaria sub-microscopic** |  |  |  |  |
| Male |  |  | Reference |  |
| Female |  |  | -2 [-5; 0] | 0.021 * |

Significance codes: < 0.001 ‘***’; < 0.01 ‘**’; < 0.05 ‘*’

**S8 Table. Household fixed-effect regression model of malaria status adjusted for age, sex, controlling for interaction between age and sex.** Number of households is 11 886, prevalence of malaria is 44% and prevalence of anemia is 83.2%.

|  | **Effect of malaria on hemoglobin** | | | |
| --- | --- | --- | --- | --- |
| **Covariate** | N | Mean Hb  [SD] [g/L] | Hb Change  [95% CI] [g/L] | P-Value |
| **Baseline total** | 17 599 | 93 [17] | Reference |  |
| **Acute effect** | 7740 | 89 [17] | -8 [-9; -7] | < 0.001 *** |
| **Prolonged effect** | 2905 | 90 [17] | -7 [-8; -6] | < 0.001 *** |
| **Sex** |  |  |  |  |
| Male | 8962 | 92 [17] | Reference |  |
| Female | 8637 | 94 [17] | 3 [1; 5] | 0.002 |
| **Age** |  |  |  |  |
| 6 - 12 | 1918 | 88 [16] | Reference |  |
| 13 - 24 | 3750 | 87 [17] | -1 [-3; 1] | 0.263 |
| 25 – 36 | 3942 | 91 [17] | 3 [2; 5] | < 0.001 *** |
| 37 – 48 | 4091 | 96 [16] | 9 [7; 10] | < 0.001 *** |
| 49 – 59 | 3898 | 110 [15] | 14 [12; 15] | < 0.001 *** |
| **Age * Female** |  |  |  |  |
| 6 – 12 |  |  | Reference |  |
| 13 – 24 |  |  | -2 [-2; 0] | 0.096 |
| 25 – 36 |  |  | -1 [-1; 2] | 0.458 |
| 37 – 48 |  |  | -1 [-1; 1] | 0.412 |
| 49 – 59 |  |  | -1 [-1; 2] | 0.556 |

Significance codes: < 0.001 ‘***’; < 0.01 ‘**’; < 0.05 ‘*’

**S9 Table. Random-effect regression model of malaria.** Number of households is 11 886, prevalence of malaria is 44% and prevalence of anemia is 83.2%.

|  | **Effect of malaria on hemoglobin** | | | |
| --- | --- | --- | --- | --- |
| **Covariate** | N | Mean Hb  [SD] [g/L] | Hb Change  [95% CI] [g/L] | P-Value |
| **Baseline total [Intercept]** | 17 599 | 93 [17] | 99 [99; 100] | < 0.001 *** |
| **Acute effect** | 7740 | 89 [17] | -10 [-11; -10] | < 0.001 *** |
| **Prolonged effect** | 2905 | 90 [17] | -9 [-10; -8] | < 0.001 *** |

Significance codes: < 0.001 ‘***’; < 0.01 ‘**’; < 0.05 ‘*’

**S10 Table. Random-effect regression model of malaria adjusted for age.** Number of households is 11 886, prevalence of malaria is 44% and prevalence of anemia is 83.2%.

|  | **Effect of malaria on hemoglobin** | | | |
| --- | --- | --- | --- | --- |
| **Covariate** | N | Mean Hb  [SD] [g/L] | Hb Change  [95% CI] [g/L] | P-Value |
| **Baseline total [Intercept]** | 17 599 | 93 [17] | 94 [93; 95] | < 0.001 *** |
| **Acute effect** | 7740 | 89 [17] | -11 [-12; -11] | < 0.001 *** |
| **Prolonged effect** | 2905 | 90 [17] | -9 [-10; -9] | < 0.001 *** |
| **Age [months]** |  |  |  |  |
| 6 – 12 | 1918 | 88 [16] | Reference |  |
| 13 – 24 | 3750 | 87 [17] | -1 [-2; 0] | 0.069 |
| 25 – 36 | 3942 | 91 [17] | 4 [3; 5] | < 0.001 *** |
| 37 – 48 | 4091 | 96 [16] | 9 [8; 1] | < 0.001 *** |
| 49 – 59 | 3898 | 110 [15] | 14 [13; 15] | < 0.001 *** |

Significance codes: < 0.001 ‘***’; < 0.01 ‘**’; < 0.05 ‘*’

**S11 Table. Random-effect regression model of malaria adjusted for sex.** Number of households is 11 886, prevalence of malaria is 44% and prevalence of anemia is 83.2%.

|  | **Effect of malaria on hemoglobin** | | | |
| --- | --- | --- | --- | --- |
| **Covariate** | N | Mean Hb  [SD] [g/L] | Hb Change  [95% CI] [g/L] | P-Value |
| **Baseline total [Intercept]** | 17 599 | 93 [17] | 98 [98; 99] |  |
| **Acute effect** | 7740 | 89 [17] | -10 [-11; -10] | < 0.001 *** |
| **Prolonged effect** | 2905 | 90 [17] | -9 [-10; -8] | < 0.001 *** |
| **Sex** |  |  |  |  |
| Male | 8962 | 92 [17] | Reference |  |
| Female | 8637 | 94 [17] | 2 [1; 2] | < 0.001 *** |

Significance codes: < 0.001 ‘***’; < 0.01 ‘**’; < 0.05 ‘*’

**S12 Table. Random-effect regression model of malaria adjusted for age and sex** Number of households is 11 886, prevalence of malaria is 44% and prevalence of anemia is 83.2%.

|  | **Effect of malaria on hemoglobin** | | | |
| --- | --- | --- | --- | --- |
| **Covariate** | N | Mean Hb  [SD] [g/L] | Hb Change  [95% CI] [g/L] | P-Value |
| **Baseline total [Intercept]** | 17 599 | 93 [17] | 93 [92; 94] | < 0.001 |
| **Acute effect** | 7740 | 89 [17] | -11 [-12; -11] | < 0.001 *** |
| **Prolonged effect** | 2905 | 90 [17] | -10 [-10; -9] | < 0.001 *** |
| **Age [months]** |  |  |  |  |
| 6 – 12 | 1918 | 88 [16] | Reference |  |
| 13 – 24 | 3750 | 87 [17] | -1 [-2; 0] | 0.072 |
| 25 – 36 | 3942 | 91 [17] | 4 [3; 5] | < 0.001 *** |
| 37 – 48 | 4091 | 96 [16] | 9 [8; 10] | < 0.001 *** |
| 49 – 59 | 3898 | 110 [15] | 14 [13; 15] | < 0.001 *** |
| **Sex** |  |  |  |  |
| Male | 8962 | 92 [17] | Reference |  |
| Female | 8637 | 94 [17] | 2 [1; 2] | < 0.001 *** |
| Model summary |  |  |  |  |

Significance codes: < 0.001 ‘***’; < 0.01 ‘**’; < 0.05 ‘*’

**S13 Table. Random-effect regression model of malaria adjusted for age and controlling for interaction between age and malaria status.** Number of households is 11 886, prevalence of malaria is 44% and prevalence of anemia is 83.2%.

|  | **Effect of malaria on hemoglobin** | | | |
| --- | --- | --- | --- | --- |
| **Covariate** | N | Mean Hb  [SD] [g/L] | Hb Change  [95% CI] [g/L] | P-Value |
| **Baseline total [Intercept]** | 17 599 | 93 [17] | 93 [92; 94] | < 0.001 *** |
| **Acute effect** | 7740 | 89 [17] | -10 [-12; -9] | < 0.001 *** |
| **Prolonged effect** | 2905 | 90 [17] | -9 [-11; -7] | < 0.001 *** |
| **Age [months]** |  |  |  |  |
| 6 – 12 | 1918 | 88 [16] | Reference |  |
| 13 – 24 | 3750 | 87 [17] | 0 [-1; 1] | 0.984 |
| 25 – 36 | 3942 | 91 [17] | 5 [4; 6] | < 0.001 *** |
| 37 – 48 | 4091 | 96 [16] | 10 [8; 11] | < 0.001 *** |
| 49 – 59 | 3898 | 110 [15] | 13 [11; 14] | < 0.001 *** |
| **Age * Malaria acute** |  |  |  |  |
| 6 – 12 |  |  | Reference |  |
| 13 – 24 |  |  | -2 [-4; 0] | 0.024 * |
| 25 – 36 |  |  | -2 [-4; 0] | 0.014 * |
| 37 – 48 |  |  | -1 [-3; 1] | 0.348 |
| 49 – 59 |  |  | 2 [0; 4] | 0.044 * |
| **Age * Malaria sub-microscopic** |  |  |  |  |
| 6 – 12 |  |  | Reference |  |
| 13 – 24 |  |  | 0 [-2; 3] | 0.926 |
| 25 – 36 |  |  | -2 [-4; 1] | 0.17 |
| 37 – 48 |  |  | -1 [-3; 1] | 0.44 |
| 49 – 59 |  |  | 1 [-1; 4] | 0.296 |

Significance codes: < 0.001 ‘***’; < 0.01 ‘**’; < 0.05 ‘*’

**S14 Table. Random-effect regression model of malaria adjusted for sex and controlling for interaction between sex and malaria status.** Number of households is 11 886, prevalence of malaria is 44% and prevalence of anemia is 83.2%.

|  | **Effect of malaria on hemoglobin** | | | |
| --- | --- | --- | --- | --- |
| **Covariate** | N | Mean Hb  [SD] [g/L] | Hb Change  [95% CI] [g/L] | P-Value |
| **Baseline total [Intercept]** | 17 599 | 93 [17] | 98 [98; 99] | < 0.001 *** |
| **Acute effect** | 7740 | 89 [17] | -10 [-11; -9] | < 0.001 *** |
| **Prolonged effect** | 2905 | 90 [17] | -9 [-10; -8] | < 0.001 *** |
| **Sex** |  |  |  |  |
| Male | 8962 | 92 [17] | Reference |  |
| Female | 8637 | 94 [17] | 2 [1; 3] | < 0.001 *** |
| **Sex * Malaria acute** |  |  |  |  |
| Male |  |  | Reference |  |
| Female |  |  | 0 [-1; 1] | 0.653 |
| **Sex * Malaria sub-microscopic** |  |  |  |  |
| Male |  |  | Reference |  |
| Female |  |  | -1 [-2; 0] | 0.184 |

Significance codes: < 0.001 ‘***’; < 0.01 ‘**’; < 0.05 ‘*’

**S15 Table. Random-effect regression model of malaria adjusted for age and sex, controlling for interaction between age and malaria status and sex and malaria status.** Number of households is 11 886, prevalence of malaria is 44% and prevalence of anemia is 83.2%.

|  | **Effect of malaria on hemoglobin** | | | |
| --- | --- | --- | --- | --- |
| **Covariate** | N | Mean Hb  [SD] [g/L] | Hb Change  [95% CI] [g/L] | P-Value |
| **Baseline total [Intercept]** | 17 599 | 93 [17] | 93 [92; 94] | < 0.001 *** |
| **Acute effect** | 7740 | 89 [17] | -10 [-12; -9] | < 0.001 *** |
| **Prolonged effect** | 2905 | 90 [17] | -9 [-11; -7] | < 0.001 *** |
| **Sex** |  |  |  |  |
| Male | 8962 | 92 [17] | Reference |  |
| Female | 8637 | 94 [17] | 2 [1; 3] | < 0.001 *** |
| **Age** |  |  |  |  |
| 6 - 12 | 1918 | 88 [16] | Reference |  |
| 13 - 24 | 3750 | 87 [17] | 0 [-1; 1] | 0.978 |
| 25 – 36 | 3942 | 91 [17] | 5 [4; 6] | < 0.001 *** |
| 37 – 48 | 4091 | 96 [16] | 10 [8; 11] | < 0.001 *** |
| 49 – 59 | 3898 | 110 [15] | 13 [11; 14] | < 0.001 *** |
| **Age * Malaria acute** |  |  |  |  |
| 6 – 12 |  |  | Reference |  |
| 13 – 24 |  |  | -2 [-4; 0] | 0.025 * |
| 25 – 36 |  |  | -2 [-4; 0] | 0.016 * |
| 37 – 48 |  |  | -1 [-3; 1] | 0.408 |
| 49 – 59 |  |  | 2 [0; 4] | 0.045 * |
| **Age * Malaria sub-microscopic** |  |  |  |  |
| 6 – 12 |  |  | Reference |  |
| 13 – 24 |  |  | 0 [-2; 3] | 0.88 |
| 25 – 36 |  |  | -2 [-4; 1] | 0.18 |
| 37 – 48 |  |  | -1 [-3; 2] | 0.491 |
| 49 – 59 |  |  | 1 [-1; 4] | 0.28 |
| **Sex * Malaria acute** |  |  |  |  |
| Male |  |  | Reference |  |
| Female |  |  | 0 [-1; 1] | 0.482 |
| **Sex * Malaria sub-microscopic** |  |  |  |  |
| Male |  |  | Reference |  |
| Female |  |  | -1 [-2; 0] | 0.143 |

Significance codes: < 0.001 ‘***’; < 0.01 ‘**’; < 0.05 ‘*’

**S16 Table. Subset analysis of females. Household fixed-effect regression of malaria status stratified by age.** Number of households is 7048, prevalence of malaria is 44.7% and prevalence of anemia is 81.8%.

|  | **Effect of malaria on hemoglobin** | | | |
| --- | --- | --- | --- | --- |
| **Covariate** | N | Mean Hb  [SD] [g/L] | Hb Change  [95% CI] [g/L] | P-Value |
| **Baseline total [Intercept]** | 8637 | 94 [17] | Reference |  |
| **Acute effect** | 3864 | 90 [17] | -6 [-8; -3] | < 0.001 *** |
| **Prolonged effect** | 1377 | 91 [17] | -6 [-9; -4] | < 0.001 *** |

Significance codes: < 0.001 ‘***’; < 0.01 ‘**’; < 0.05 ‘*’

**S17 Table. Subset analysis of females. Household fixed-effect regression of malaria status adjusted for age.** For thick smear microscopy: number of households is 7052, prevalence of malaria is 44.7% and prevalence of anemia is 81.8%. For rapid diagnostic test: number of households is 7048, prevalence of malaria is 54.6%, prevalence of anemia is 81.8%. We excluded an additional 9 children from rapid diagnostic test analysis because no outcome data was available.

|  | **Effect of malaria on hemoglobin** | | | |
| --- | --- | --- | --- | --- |
| **Covariate** | N | Mean Hb  [SD] [g/L] | Hb Change  [95% CI] [g/L] | P-Value |
| **Baseline total [Intercept]** | 8637 | 94 [17] | Reference |  |
| **Acute effect** | 3864 | 90 [17] | -9 [-11; -7] | < 0.001 *** |
| **Prolonged effect** | 1377 | 91 [17] | -9 [-12; -7] | < 0.001 *** |
| **Age [months]** |  |  |  |  |
| 6 – 12 | 934 | 89 [16] | Reference |  |
| 13 – 24 | 1805 | 88 [17] | -3 [-6; 1] | 0.01 * |
| 25 – 36 | 1910 | 92 [17] | 2 [0; 5] | 0.09 |
| 37 – 48 | 2071 | 9.7 [16] | 8 [6; 1] | < 0.001 *** |
| 49 – 59 | 1917 | 110 [15] | 12 [10; 15] | < 0.001 *** |

Significance codes: < 0.001 ‘***’; < 0.01 ‘**’; < 0.05 ‘*’

**S18 Table. Subset analysis of females. Household fixed-effect regression of malaria status adjusted for age and controlling for interaction between age and malaria status.** For thick smear microscopy: number of households is 7052, prevalence of malaria is 44.7% and prevalence of anemia is 81.8%. For rapid diagnostic test: number of households is 7048, prevalence of malaria is 54.6%, prevalence of anemia is 81.8%. We excluded an additional 9 children from rapid diagnostic test analysis because no outcome data was available.

|  | **Effect of malaria on hemoglobin** | | | |
| --- | --- | --- | --- | --- |
| **Covariate** | N | Mean Hb  [SD] [g/L] | Hb Change  [95% CI] [g/L] | P-Value |
| **Baseline total [Intercept]** | 8637 | 94 [17] | Reference |  |
| **Acute effect** | 3864 | 90 [17] | -10 [-14; -5] | < 0.001 *** |
| **Prolonged effect** | 1377 | 91 [17] | -8 [-13; -2] | < 0.005 ** |
| **Age [months]** |  |  |  |  |
| 6 – 12 | 934 | 89 [16] | Reference |  |
| 13 – 24 | 1805 | 88 [17] | -1 [-5; 3] | 0.729 |
| 25 – 36 | 1910 | 92 [17] | 2 [-2; 6] | 0.261 |
| 37 – 48 | 2071 | 9.7 [16] | 9 [5; 12] | < 0.001 *** |
| 49 – 59 | 1917 | 110 [15] | 9 [6; 13] | < 0.001 *** |
| Age * malaria acute |  |  |  |  |
| 6 – 12 |  |  | Reference |  |
| 13 – 24 |  |  | -3 [-8; 3] | 0.298 |
| 25 – 36 |  |  | 0 [-5; 6] | 0.957 |
| 37 – 48 |  |  | 0 [-5; 5] | 0.94 |
| 49 – 59 |  |  | 5 [0; 1] | 0.045 * |
| Age * malaria chronic |  |  |  |  |
| 6 – 12 |  |  | Reference |  |
| 13 – 24 |  |  | -6 [-13; 1] | 0.116 |
| 25 – 36 |  |  | 0 [-7; 7] | 0.975 |
| 37 – 48 |  |  | -3 [-9; 4] | 0.418 |
| 49 – 59 |  |  | 3 [-4; 9] | 0.43 |

Significance codes: < 0.001 ‘***’; < 0.01 ‘**’; < 0.05 ‘*’

**S19 Table. Subset analysis of males. Household fixed-effect regression of malaria status.** Number of households is 7215, prevalence of malaria is 43.2% and prevalence of anemia is 84.5%.

|  | **Effect of malaria on hemoglobin** | | | |
| --- | --- | --- | --- | --- |
| **Covariate** | N | Mean Hb  [SD] [g/L] | Hb Change  [95% CI] [g/L] | P-Value |
| **Baseline total [Intercept]** | 8962 | 92 [17] | Reference |  |
| **Acute effect** | 3876 | 88 [17] | -4 [-6; -1] | 0.002 ** |
| **Prolonged effect** | 1528 | 89 [16] | -4 [-6; -1] | 0.002 ** |

Significance codes: < 0.001 ‘***’; < 0.01 ‘**’; < 0.05 ‘*’

**S20 Table. Subset analysis of males. Household fixed-effect regression of malaria status adjusted for age.** Number of households is 7215, prevalence of malaria is 43.2% and prevalence of anemia is 84.5%.

|  | **Effect of malaria on hemoglobin** | | | |
| --- | --- | --- | --- | --- |
| **Covariate** | N | Mean Hb  [SD] [g/L] | Hb Change  [95% CI] [g/L] | P-Value |
| **Baseline total [Intercept]** | 8962 | 92 [17] | Reference |  |
| **Acute effect** | 3876 | 88 [17] | -7 [-9; -5] | < 0.001 *** |
| **Prolonged effect** | 1528 | 89 [16] | -5 [-8; -3] | < 0.001 *** |
| **Age [months]** |  |  |  |  |
| 6 – 12 | 984 | 87 [17] | Reference |  |
| 13 – 24 | 1945 | 86 [17] | -2 [-4; 1] | 0.128 |
| 25 – 36 | 2032 | 90 [17] | 3 [1; 5] | 0.01 * |
| 37 – 48 | 2020 | 95 [17] | 8 [5; 1] | < 0.001 *** |
| 49 – 59 | 1981 | 10 [16] | 13 [11; 16] | < 0.001 *** |

Significance codes: < 0.001 ‘***’; < 0.01 ‘**’; < 0.05 ‘*’

**S21 Table. Subset analysis of males. Household fixed-effect regression of malaria status adjusted for age and controlling for interaction between age and malaria status.** Number of households is 7215, prevalence of malaria is 43.2% and prevalence of anemia is 84.5%.

|  | **Effect of malaria on hemoglobin** | | | |
| --- | --- | --- | --- | --- |
| **Covariate** | N | Mean Hb  [SD] [g/L] | Hb Change  [95% CI] [g/L] | P-Value |
| **Baseline total [Intercept]** | 8962 | 92 [17] | Reference |  |
| **Acute effect** | 3876 | 88 [17] | -5 [-9; -1] | 0.017 * |
| **Prolonged effect** | 1528 | 89 [16] | -3 [-8; 2] | 0.309 |
| **Age [months]** |  |  |  |  |
| 6 – 12 | 984 | 87 [17] | Reference |  |
| 13 – 24 | 1945 | 86 [17] | 1 [-2; 5] | 0.479 |
| 25 – 36 | 2032 | 90 [17] | 7 [3; 11] | < 0.001 *** |
| 37 – 48 | 2020 | 95 [17] | 8 [4; 11] | < 0.001 *** |
| 49 – 59 | 1981 | 10 [16] | 13 [10; 16] | < 0.001 *** |
| Age * malaria acute |  |  |  |  |
| 6 – 12 |  |  | Reference |  |
| 13 – 24 |  |  | -7 [-12; -2] | 0.01 * |
| 25 – 36 |  |  | -6 [-11; 0] | 0.032 * |
| 37 – 48 |  |  | 0 [-5; 5] | 0.901 |
| 49 – 59 |  |  | 1 [-4; 6] | 0.719 |
| Age * malaria chronic |  |  |  |  |
| 6 – 12 |  |  | Reference |  |
| 13 – 24 |  |  | -3 [-9; 4] | 0.389 |
| 25 – 36 |  |  | -7 [-14; -1] | 0.072 |
| 37 – 48 |  |  | -3 [-1; 3] | 0.338 |
| 49 – 59 |  |  | -1 [-7; 6] | 0.842 |

Significance codes: < 0.001 ‘***’; < 0.01 ‘**’; < 0.05 ‘*’

**S22 Table. Subset analysis of participants tested during the low malaria season (December – June). Household fixed-effect regression main model of malaria status adjusted for age and controlling for interaction between age and malaria status.** Number of households is 4398, prevalence of malaria is 23.5% and prevalence of anemia is 76.3%. Included surveys are MIS 2010-11 (975), MIS 2014 (3), MIS 2017-18 (5415).

|  | **Effect of malaria on hemoglobin** | | | |
| --- | --- | --- | --- | --- |
| **Covariate** | N | Mean Hb  [SD] [g/L] | Hb Change  [95% CI] [g/L] | P-Value |
| **Baseline total** | 6393 | 98 [16] | Reference |  |
| **Acute effect** | 1499 | 93 [16] | -7 [-9; -6] | < 0.001 *** |
| **Prolonged effect** | 613 | 93 [16] | -7 [-1; -5] | < 0.001 *** |
| **Sex** |  |  |  |  |
| Male | 3241 | 98 [16] | Reference |  |
| Female | 3152 | 99 [16] | 2 [1; 3] | 0.002 |
| **Age** |  |  |  |  |
| 6 - 12 | 708 | 104 [15] | Reference |  |
| 13 - 24 | 1326 | 92 [16] | -2 [-4; 0] | 0.126 |
| 25 – 36 | 1397 | 98 [16] | 4 [2; 6] | < 0.001 *** |
| 37 – 48 | 1520 | 110 [15] | 7 [5; 9] | < 0.001 *** |
| 49 – 59 | 1442 | 104 [14] | 12 [10; 14] | < 0.001 *** |

Significance codes: < 0.001 ‘***’; < 0.01 ‘**’; < 0.05 ‘*’

**S23 Table. Subset analysis of participants tested during the peak malaria season (July – November). Household fixed-effect regression of malaria status adjusted for age and controlling for interaction between age and malaria status.** For thick smear microscopy: number of households is 7317, prevalence of malaria is 55.7% and prevalence of anemia is 87.1%. Included surveys are MIS 2010-11 (4951), MIS 2014 (6107), MIS 2017-18 (148).

|  | **Effect of malaria on hemoglobin** | | | |
| --- | --- | --- | --- | --- |
| **Covariate** | N | Mean Hb  [SD] [g/L] | Hb Change  [95% CI] [g/L] | P-Value |
| **Baseline total** | 11 206 | 90 [17] | Reference |  |
| **Acute effect** | 6241 | 88 [17] | -8 [-9; -6] | < 0.001 *** |
| **Prolonged effect** | 2292 | 89 [17] | -7 [-8; -5] | < 0.001 *** |
| **Sex** |  |  |  |  |
| Male | 5721 | 89 [17] | Reference |  |
| Female | 5485 | 91 [17] | 2 [1; 3] | < 0.001 *** |
| **Age** |  |  |  |  |
| 6 - 12 | 1210 | 86 [16] | Reference |  |
| 13 - 24 | 2424 | 84 [17] | -2 [-4; -1] | 0.008 ** |
| 25 – 36 | 2545 | 87 [17] | 2 [0; 4] | 0.013 * |
| 37 – 48 | 2571 | 93 [16] | 9 [7; 10] | < 0.001 *** |
| 49 – 59 | 2456 | 98 [16] | 14 [13; 16] | < 0.001 *** |

Significance codes: < 0.001 ‘***’; < 0.01 ‘**’; < 0.05 ‘*’

**S24** **Subset analysis of MIS 2010-11 (May – January). Household fixed-effect regression of malaria status adjusted for age and controlling for interaction between age and malaria status.** The number of households is 3939, prevalence of malaria is 65.2% and prevalence of anemia is 88.2%. Data was gathered during high season (4951) and low season (975).

|  | **Effect of malaria on hemoglobin** | | | |
| --- | --- | --- | --- | --- |
| **Covariate** | N | Mean Hb  [SD] [g/L] | Hb Change  [95% CI] [g/L] | P-Value |
| **Baseline total** | 5926 | 90 [17] | Reference |  |
| **Acute effect** | 3861 | 89 [17] | -2 [-4; 0] | 0.059 |
| **Prolonged effect** | 1020 | 90 [17] | 0 [-3; 2] | 0.787 |
| **Sex** |  |  |  |  |
| Male | 3044 | 90 [17] | Reference |  |
| Female | 2882 | 91 [17] | 2 [1; 3] | 0.003 ** |
| **Age** |  |  |  |  |
| 6 - 12 | 651 | 85 [17] | Reference |  |
| 13 - 24 | 1301 | 84 [16] | -1 [-3; 1] | 0.226 |
| 25 – 36 | 1328 | 88 [16] | 2 [0; 4] | 0.062 |
| 37 – 48 | 1363 | 93 [15] | 9 [8; 11] | < 0.001 *** |
| 49 – 59 | 1283 | 99 [15] | 16 [14; 18] | < 0.001 *** |

Significance codes: < 0.001 ‘***’; < 0.01 ‘**’; < 0.05 ‘*’

**S25** **Subset analysis of MIS 2014 (September – December).** The number of households is 3938, prevalence of malaria is 47.7% and prevalence of anemia is 86.6%. Data was gathered during high season (6107) and low season (3).

|  | **Effect of malaria on hemoglobin** | | | |
| --- | --- | --- | --- | --- |
| **Covariate** | N | Mean Hb  [SD] [g/L] | Hb Change  [95% CI] [g/L] | P-Value |
| **Baseline total** | 6110 | 90 [17] | Reference |  |
| **Acute effect** | 2913 | 86 [17] | -11 [-12; -9] | < 0.001 *** |
| **Prolonged effect** | 1787 | 89 [17] | -10 [-12; -8] | < 0.001 *** |
| **Sex** |  |  |  |  |
| Male | 3107 | 89 [18] | Reference |  |
| Female | 3003 | 91 [17] | 2 [1; 3] | < 0.001 *** |
| **Age** |  |  |  |  |
| 6 - 12 | 657 | 87 [16] | Reference |  |
| 13 - 24 | 1297 | 84 [17] | -2 [-4; 0] | 0.048 * |
| 25 – 36 | 1404 | 87 [16] | 2 [0; 5] | 0.038 * |
| 37 – 48 | 1402 | 94 [17] | 8 [6; 10] | < 0.001 *** |
| 49 – 59 | 1350 | 98 [16] | 13 [11; 15] | < 0.001 *** |

Significance codes: < 0.001 ‘***’; < 0.01 ‘**’; < 0.05 ‘*’

**S26** **Subset analysis of MIS 2017-18 (November – March).** The number of households is 3838, prevalence of malaria is 17.4% and prevalence of anemia is 74%. Data was gathered during high season (148) and low season (5415).

|  | **Effect of malaria on hemoglobin** | | | |
| --- | --- | --- | --- | --- |
| **Covariate** | N | Mean Hb  [SD] [g/L] | Hb Change  [95% CI] [g/L] | P-Value |
| **Baseline total** | 5563 | 99 [16] | Reference |  |
| **Acute effect** | 966 | 95 [16] | -8 [-10; -6] | < 0.001 *** |
| **Prolonged effect** | 475 | 93 [16] | -8 [-11; -6] | < 0.001 *** |
| **Sex** |  |  |  |  |
| Male | 2811 | 99 [16] | Reference |  |
| Female | 2752 | 10 [15] | 2 [1; 3] | 0.002** |
| **Age** |  |  |  |  |
| 6 - 12 | 610 | 94 [14] | Reference |  |
| 13 - 24 | 1152 | 93 [15] | -2 [-5; 0] | 0.035 * |
| 25 – 36 | 1210 | 99 [15] | 4 [2; 7] | < 0.001 *** |
| 37 – 48 | 1326 | 102 [15] | 7 [5; 9] | < 0.001 *** |
| 49 – 59 | 1265 | 105 [14] | 11 [9; 13] | < 0.001 *** |

Significance codes: < 0.001 ‘***’; < 0.01 ‘**’; < 0.05 ‘*’

*
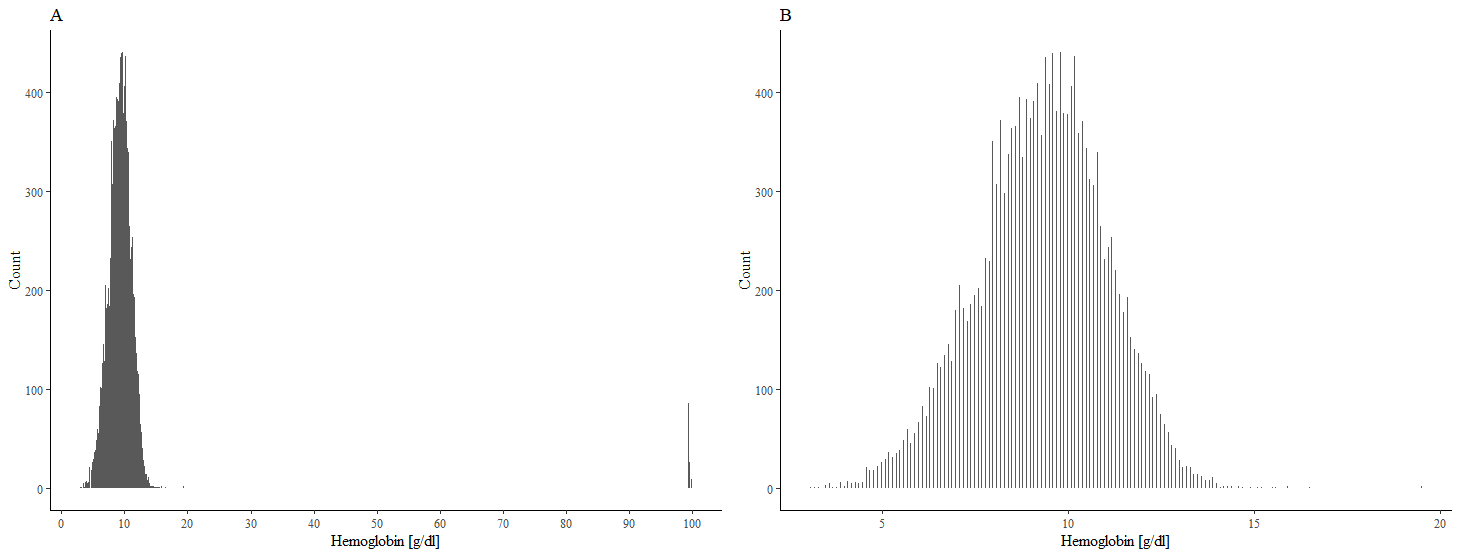
*

**S27 Histograms of the hemoglobin distribution in the study population before (A) and after (B) excluding values of > 50 g/dL.** We excluded 176 cases with hemoglobin values from 994 to 999 g/dL from the analysis which left the distribution as shown in panel B.


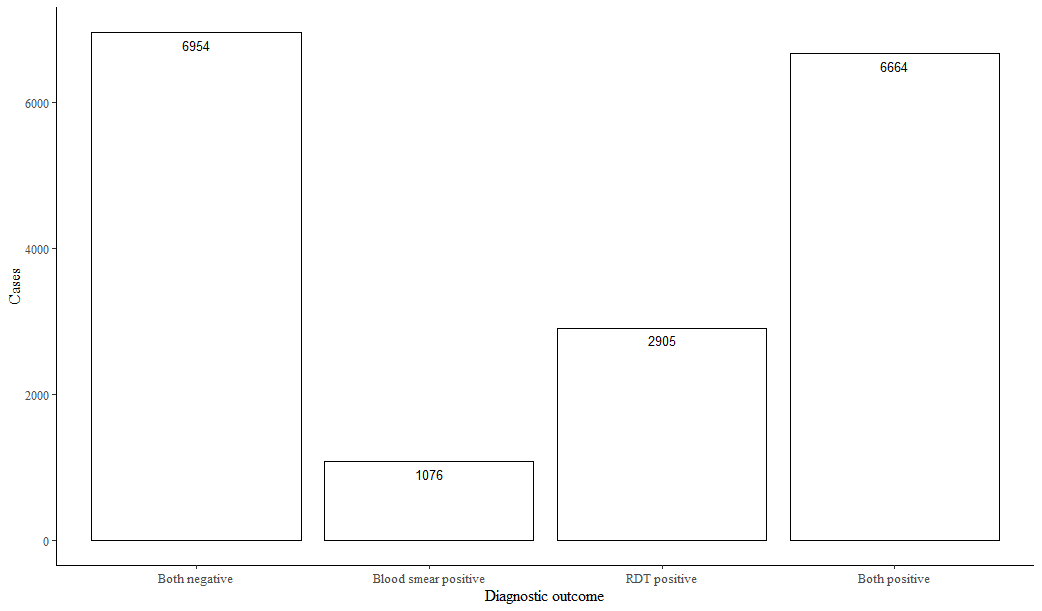


**S28 Disparities in diagnostic outcome based on test method.** Overlap between thick smear microscopy results and rapid diagnostic test results is somewhat limited. This can be a result of limited test quality and the time course of disease.

**S29. Final model form and internal parameters:**

The final model was stratified for malaria status, age, sex and household fixed-effect and takes the following form:

*Y_im_= α + β_V_V_im_ + β_X_X_im_ + β_W_W_im_ + μ_m_ + ε_im_*

where

*α* is the intercept,

*Y_im_* is the Hb concentration of child *i* within household *m,*

*V_im_* is the child *i’s* Malaria status,

*X_im_* is the child *i*’s age group,

*W_im_* is the child *i*’s sex,

*β_V/X/W_* is the association between Hb concentration and Malaria status/age group/sex,

*μ_m_* is the household fixed effect and

*ɛ_im_* is the residual unexplained individual-level variation.


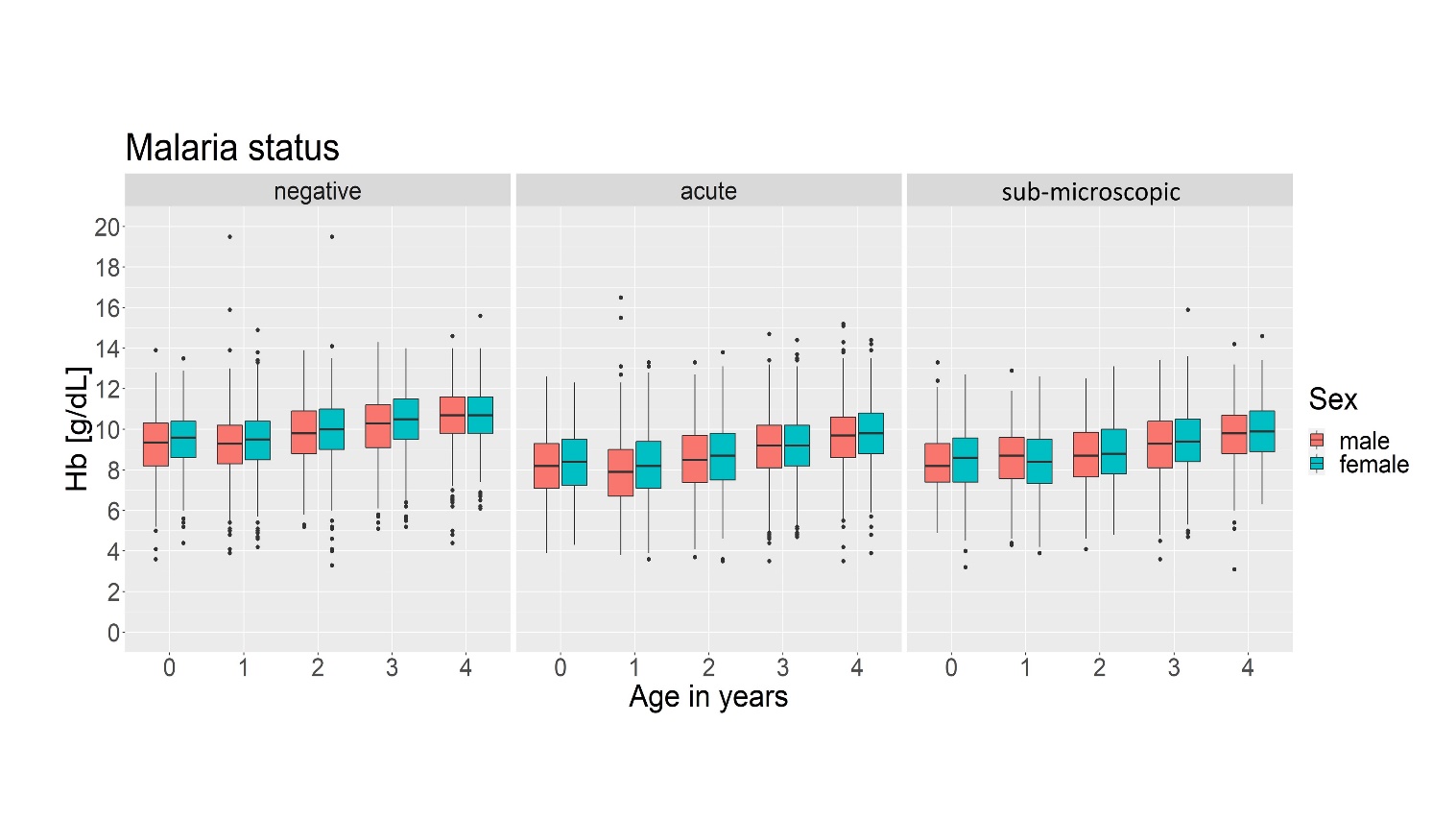


**S30. Haemoglobin distribution based on malaria status and sex by age group.** In both boys and girls, hemoglobin levels increase with age. Overall, girls have slightly higher mean hemoglobin than boys. Malaria positive children have lower hemoglobin levels throughout all age groups.
